# Lower Limb Tendinopathy Tissue Changes Assessed through Ultrasound: A Narrative Review

**DOI:** 10.3390/medicina56080378

**Published:** 2020-07-28

**Authors:** Eleuterio A. Sánchez Romero, Joel Pollet, Sebastián Martín Pérez, José Luis Alonso Pérez, Alberto Carlos Muñoz Fernández, Paolo Pedersini, Carlos Barragán Carballar, Jorge Hugo Villafañe

**Affiliations:** 1 Musculoskeletal Pain and Motor Control Research Group, Faculty of Health Sciences, Universidad Europea de Madrid, 28670 Madrid, Spain; joseluis.alonso@universidadeuropea.es (J.L.A.P.); albertocarlos.munoz@universidadeuropea.es (A.C.M.F.); carlos.barragan.carballar@gmail.com (C.B.C.); 2 Department of Physiotherapy, Faculty of Biomedical and Health Sciences, Universidad Europea de Madrid, Tajo, s/n, Urbanización El Bosque, 28670 Villaviciosa de Odón, Madrid, Spain; sebastian.martin@universidadeuropea.es; 3 IRCCS Fondazione Don Carlo Gnocchi, 20161 Milan, Italy; joel.pollet.ft@gmail.com (J.P.); pedersini93@gmail.com (P.P.); mail@villafane.it (J.H.V.); 4 Musculoskeletal Pain and Motor Control Research Group, Faculty of Health Sciences, Universidad Europea de Canarias, C/Inocencio García 1 38300 La Orotava, 38300 Tenerife, Canary Islands, Spain

**Keywords:** tendinopathy, ultrasonography, reliability

## Abstract

Tendinopathy is a common disease that affects athletes, causing pain and dysfunction to the afflicted tendon. A clinical diagnose is usually combined with imaging and, among all the existing techniques, ultrasound is widely adopted. The aim of this review is to sum up the existing evidence on ultrasound as an imaging tool and guide for treatments in lower limbs tendinopathy. Using three different databases—PubMed, MEDLINE and CENTRAL—a literature search has been performed in May 2020 combining MeSH terms and free terms with Boolean operators. Authors independently selected studies, conducted quality assessment, and extracted results. Ultrasound imaging has a good reliability in the differentiation between healthy and abnormal tendon tissue, while there are difficulties in the identification of tendinopathy stages. The main parameters considered by ultrasound imaging are tendon thickness, hypoechogenicity of tendon structure and neovascularization of the tendon bound tissue. Ultrasound-guide is also used in many tendinopathy treatments and the available studies gave encouraging results, even if further studies are needed in this field.

## 1. Introduction

Soft tissue injuries of the lower limb are widely diffuse and put a significant financial burden on the health care systems worldwide. Tendinopathy is a common problem in the adult population that especially affects elite athletes and amateurs. The prevalence of tendinopathies in sporting people is 22%, with differences related to sport and to the level [1]. Patellar tendinopathy, one of the commonest tendinopathy, afflicts the 45% of elite volleyball players and the 32% of elite basketball players [2], in elite soccer players, cumulative trauma disorder and re-injuries constituted 37% and 22% of all injuries [3], while among non-elite players of all the different sports, the incidence is lower, only 14%, but still remarkable [4]. While the prevalence in elite athletes of the Achilles tendinopathy is 5%, and rises till 9% in recreational runners [5]. Quadriceps tendinopathy prevalence is about 14.2% of elite athletes, especially in sports that require repetitive jumps [2]. Proximal Hamstring Tendinopathy and Gluteus tendinopathy are most common among distance runners and athletes performing, but their prevalence is still nuclear [6,7].

Abnormal kinematics and the overuse of the tendon have been implicated as the major risk factors for lower limb tendinopathy, even if the amount of loading that generates the pathology is still not clear [8,9]. Overuse is a key factor from 30% to 50% of all sporting injuries and the incidence has raised in recent decades, likely due to the growing involvement on athletes and greater demands in running and recreational sports. For what concerns the pathogenesis, different theories have been proposed [10]: (i) degenerative theory proposes that overloading causes changes in tendon cells and degeneration of the matrix [11]; (ii) failed healing theory suggests that, at the early stage of tendinopathy, a healing process that increases the production of protein is ongoing and causes a disorganisation of the matrix [12,13]; (iii) unloading theory suggests that not only overloading causes the changes in the cell and matrix of the tendon, but also unloading [14]; (iv) the last and widely accepted theory is the continuum theory, in which tendon pathology is composed by three stages (reactive tendinopathy, tendon disrepair and degenerative tendinopathy) in continuity between them [15].

Therefore, to better understand the clinical condition of a subject that presents signs of tendinopathy, imaging gains particular importance. In this context, different imaging tools have been proposed to evaluate the condition of the tendon structure. Radiographs and computer tomography are mainly used to evaluate arthritis and calcific tendinitis [10,16]. However, the most diffused imaging tools for tendinopathy are magnetic resonance imaging (MRI) and ultrasound. Many studies compared these imaging tools, but, to date, a gold standard between them has not been identified. Their benefits and harms have been deeply studied by many authors [17,18]. The main advantage of ultrasound is the cost-effectiveness compared to MRI and flexibility of setting in which it can be used, moreover, it is demonstrated that ultrasound allows a higher spatial resolution than MRI thanks to modern high-frequency transducers (10–15 Mhz) [19], and the main disadvantage is represented by the accuracy that is operator-dependent. Another point not to forget is that ultrasound is used also to assess ultrasound-guide treatments, whose use has been widely adopted in many fields of medicine, such as catheterization in cancer patients [20], stem cell transplantation [21] and local anaesthetic infiltration [22]. Ultrasound became popular for its advantages, such as being minimally invasive, fast, easy to use and controlling the effectiveness of treatments.

The aim of this review is to summarise and analyse the role of ultrasound imaging as diagnostic tool and as a treatment guide for the management of lower limb tendinopathies.

## 2. Methods

Authors performed a literature search to identify all the available studies published from their inception to May 2010 that evaluated ultrasound as an imaging tool and as guide for treatments for lower limbs tendinopathy. In this review, a literature research was performed using three different databases: PubMed, MEDLINE and CENTRAL. The search strategies used MeSH terms and free terms combined with Boolean operators *AND, OR, NOT*. The MeSH terms used were: tendons, tendinopathy, lower extremity, ultrasonography. The free terms used were: “lower limb”, tendon, “Achilles tendon”, “patellar tendon”, “quadriceps tendon”, “hamstring tendon”, “gluteus tendon”, “tendinopathy”, “ultrasonography”. The literature search was performed to identify all the available studies published from their inception to the 1st May 2020. Authors independently selected studies, conducted quality assessments, and extracted the results. The methodological quality of the RCTs included was acceptable with a mean total score of 7.37 on the PEDro scale. The Newcastle-Ottawa Scale (NOS) for assessing the methodological quality of the nonrandomized studies included had an average of 4.25 total score, representing medium to high quality.

**Figure 1:**
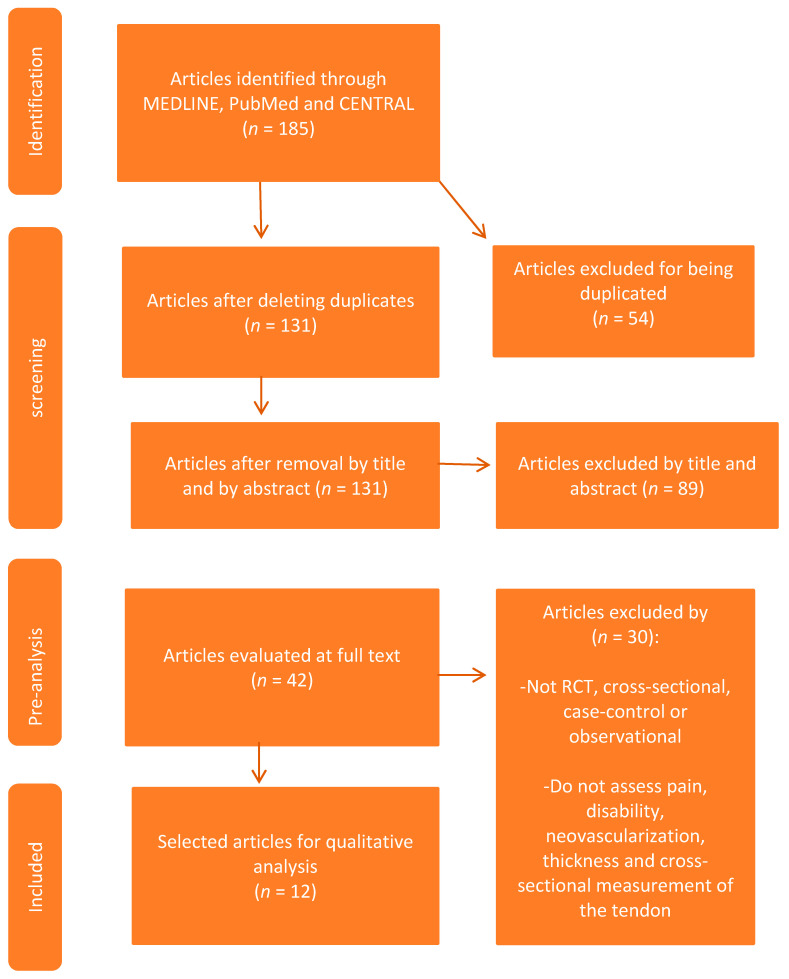
Selection and data extraction procedure.

The flow diagram of the selection and data extraction process is presented in Figure 1.

## 3. Result

**Figure 2:**
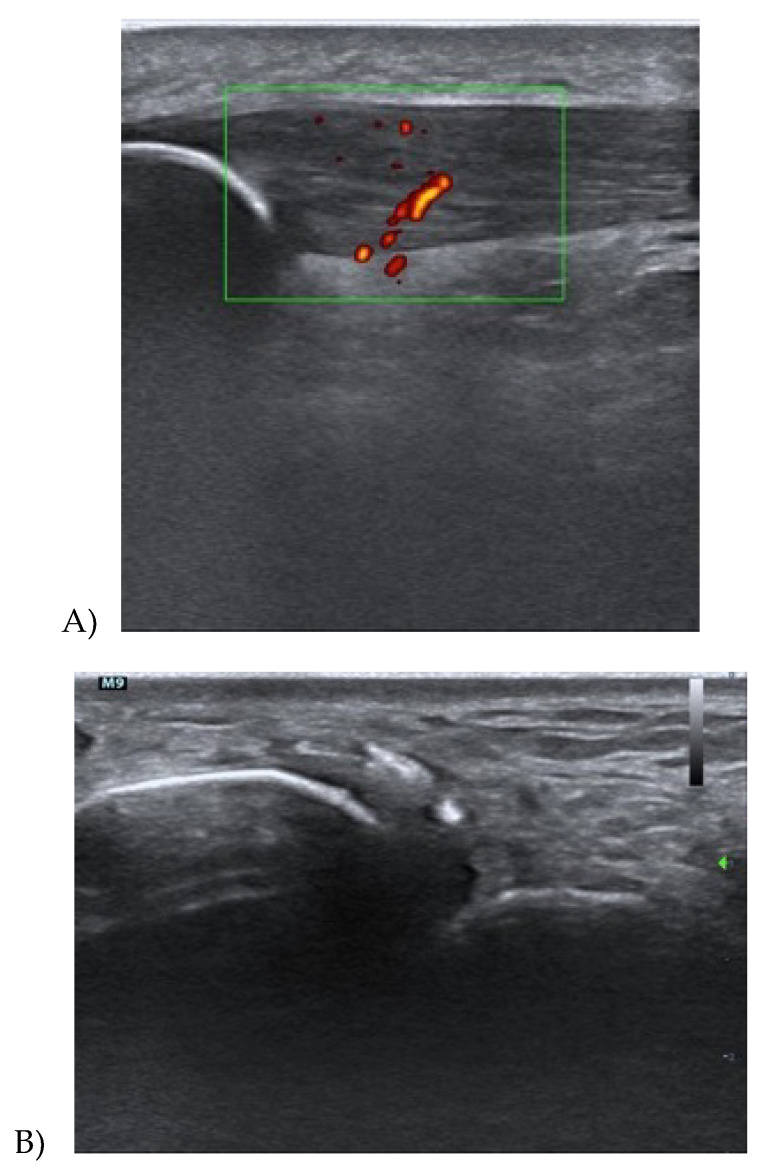
(**A**,**B**) Longitudinal section of the patellar tendon and the Achilles tendon showing the considers the primary 3 tendinopathy features: thickness, hypoechogenicity of tendon tissue, and neovascularization. The green box corresponds to the area of the Power Doppler function. The green arrow corresponds to the focus area.

**Figure 3:**
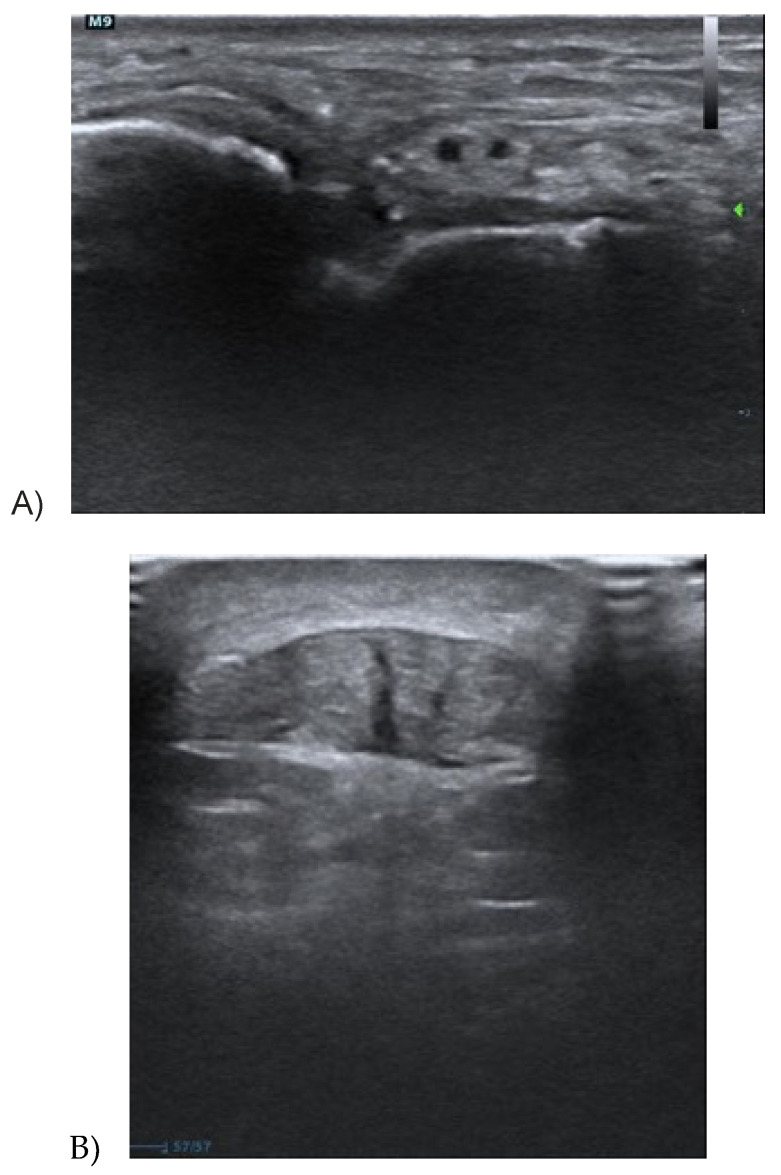
(**A**,**B**) Longitudinal and cross section of the patellar tendon and the Achilles tendon showing hypoechogenicity and collagen fibrils disorganization. The green arrow corresponds to the focus area.

Tendinopathy has primarily a clinical diagnosis, but it is usually combined with imaging. Ultrasound is the commonest examination for tendons and it considers primary three tendon features: (i) Thickness—tendinopathy can cause an increase in tendon thickness, due to a change in the number and type of cells of the tendon tissue, and this event provokes a bound water increase, and consequently an augmented tendon dimension [13]. Tendon thickness is indeed moderately correlated with pain, for some authors [23,24,25,26], so it is considered as an indirect measure of treatment outcome [8,27]. Specifically, Romero C et al. [28] found that an increase in Achilles tendon thickness and its cross-sectional area at 4 and 6 cm from the calcaneus, comparing subjects with and without Achilles tendinopathy. (ii) The hypoechogenicity of tendon tissue is due to a change in collagen fibre type, from type I, in healthy tendons, to type II and III in pathological tendons. The ultrasound alteration is caused by the disorganization of collagen fibres [29]. The initial stage of a tendinopathy presents small focal areas of hypoechogenicity, in discontinuity with the normal tendon tissue, while in the worst cases, entire regions of the tendon present this kind of alteration [13]. (iii) Neovascularization is associated with hypoechogenicity areas and represents an increase in blood vessels in the area nearby the tendon. It is identified through colour or power Doppler. These techniques show a greater number of blood vessels in the case of tendon tissue alterations [13]; Figure 2 and Figure 3.

Many studies tried to correlate these outcomes with pain and disability, but, in most cases, this was not possible [22,30]. It is also important to highlight that imaging alterations have to be interpreted within the clinical examination of pain and function, because there is a weak relationship between any kind of imaging and pain [17]; indeed, there are many cases of tendon alterations retrieved through imaging without pain or dysfunction. On the contrary, a recent review has shown that alterations in at least two of the ultrasound parameters for tendon have a relative risk (RR) of 3.66 to develop symptomatic tendinopathy, while the combination of three parameters has the relative risk of developing symptoms of 6.49 [31]. These considerations highlight the importance of ultrasound as a fundamental tool to assist the clinician in the diagnosis of lower limb tendinopathies through the evaluation of the three fundamental parameters of tendon structure.

### 3.1. Achilles Tendinopathy

This is considered a common condition in athletes. The clinical diagnosis is associated with ultrasound, and numerous studies showed that it has a good intra- and inter-rater reliability [23,32,33].

Some studies adopted a new technique called ultrasound tissue characterization (UTC) that reconstructs the tendon structure in 3D and stages the tendon in four echo types (I–IV) [34]. This technique has an excellent reliability (0.92–0.95) as shown by Van Schie et al. [35]. UTC has demonstrated to be effective in the evaluation of tendinopathy and to assess the tendon improvements [36,37,38].

Matthews et al. [39] have recently proposed a new method to investigate Achilles tendinopathy with ultrasound, using the continuum model of tendinopathy development [12]. The results showed a moderate to excellent intra- and inter-rater reliability of the overall outcome (0.52–0.99). Further studies should confirm this result.

### 3.2. Patellar Tendinopathy

This tendinopathy is also known as jumper knee. The ultrasound in the evaluation process of this kind of tendinopathy is widely adopted [40] since the first studies of Cook et al. that showed the prevalence of this kind of pathology in basketball players and athletes with jump activities [41,42]. Ultrasounds have a good reliability in the identification of the common alterations affecting the tendon [23]. In particular, as for Achilles tendinopathy, UTC technique has been adopted for patellar tendinopathy. Van Ark et al. showed an intra-rater reliability between 0.80–0.93, and an inter-rater reliability among 0.71–0.90 [43]; these encouraging results should be confirmed by other studies, but adopting these techniques should be considered as an outcome measure for studies on the effectiveness of treatments for tendinopathy.

### 3.3. Quadriceps Tendinopathy

This is a less common tendinopathy then patellar tendinopathy; it affects the cranial part of the patella. It is considered as part of the jumper knee syndrome and usually it is not differentiated from the patellar tendinopathy, while, as highlighted by Sprague et al., the two tendons have different structures and different treatments [44]. Quadriceps tendinopathy, due to its low prevalence, has rarely been studied with ultrasound [45]. Future studies should focus on this particular condition, differentiating it from the most common patellar tendinopathy.

### 3.4. Proximal Hamstring Tendinopathy

This is also called high hamstring tendinopathy, and is an uncommon hamstring injury, that causes pain in the posterior upper part of thigh. It has a clinical diagnosis that is usually combined with imaging due to the differential diagnosis that could cause the symptoms [46]. Few studies considered ultrasound [47] and no studies compared the reliability of this imaging tool.

### 3.5. Gluteus Tendinopathy

This includes tendinopathies at the different gluteus muscles, maximus, medius and minimus. Regarding the gluteus maximus, it is usually interested by calcific tendinitis but, for diagnosis, computer tomography is the gold standard [16]. Gluteus medius and minimus tendinopathy, instead, is a common condition that gave lateral hip pain and has a differential diagnosis with many conditions. Connell et al. showed that, in most cases, lateral hip pain is associated with ultrasound alterations to the gluteus medius and minimus tendon [48]. Other studies confirmed this hypothesis [49]. The ability to differentiate between a healthy and a pathological medius gluteus tendon has recently been tested, with encouraging results, while the differentiation of the different tendinopathy stages was poor [50]. Further studies should consider the newly techniques used for the ultrasound imaging of other tendons to test the tendinopathy stages.

### 3.6. Treatments Using Ultrasound Imaging

Ultrasound imaging has been used also in the treatment of tendinopathy. Ultrasound-guided interventions are performed to treat tendinopathies of lower and upper limbs with different kinds of intervention [51]. The ultrasound-guided injection of different solutions of Platelet-Rich Plasma or corticosteroid have been studied and performed on different tendons in the lower limb [51,52,53]. Another common intervention that uses an ultrasound guide is the tendon tenotomy, which exploits high-frequency energy to remove the pathological tissue and stimulates an acute inflammatory process that should help healthy tendon tissue to grow. This intervention is performed in the different tendinopathies of the lower limb [54,55]. Another interesting ultrasound-guided intervention for tendinopathies is dry needling, which uses a repeated needle to stimulate an inflammatory process in the abnormal tendon tissue and, through the granulation process of inflammation strength, the tendon tissue [56]. This intervention has been adopted in different clinical settings. However, the effects on tendinopathies need to be studied with high-quality studies, because of the limitation of this topic in the literature [57].

## 4. Discussion

The literature shows how ultrasound imaging is widely adopted in the diagnostic phase of many lower limb tendinopathies. Ultrasound is also used as a key tool to perform guided interventions in the pathological areas of tendinopathy. Possible areas of interest have been highlighted for future research on uncommon lower limb tendinopathies, using ultrasound as an imaging tool and as a treatment guide. These will improve the clinical diagnose and management of these tricky conditions.

As previously said, the role of the clinician is fundamental in the diagnosis of tendinopathy, and ultrasonography is the tool that allows the identification of the typical pathological features of tendinopathy. These important signs are widely used by researchers and clinicians. The reliability of ultrasound as an imaging tool has been the major problem for these technique, a systematic review on shoulder tendinopathy paradoxically showed similar sensitivity and specificity [58], the study of Warden et al. [59] instead showed higher values of sensibility and specificity with respect to MRI for patellar tendinopathies, similar studies should be performed also on lower limb tendon imaging. Newly developed ultrasound methods like UTC have shown to be even more reliable and precise then ultrasound itself. The quality of these studies is conditioned by their study design (observational studies), but the sample included in some of those [34,43] makes their results of high value. A comparison of these technique with other imaging tools will display the real value of this technique.

The current literature did not provide a shared opinion on the possible correlation between the tendon ultrasound alterations and pain. Some points can be defined; it is possible to have tendon alteration without pain [18], but the alteration of tendon structure has a high RR to develop tendinopathy in the future [32]. The studies of many authors gave different results, some showed a good correlation among pain and thickness [23,25], other showed higher correlation between pain and neovascularization [24,30]. The results presented at the International Scientific Tendinopathy Symposium [22], instead suggests a weak correlation and to consider the results of ultrasound carefully in the evaluation of patients due to a lack of imaging improvement in the short term. The studies produced did not present experimental designs, this represents an issue, but the sample considered gave the result of these studies a good value. The use of experimental designs to verify the hypothesis of a correlation will give the field a sure impact, also using the UTC technology [22].

The ultrasound-guided treatments are relatively young treatments; the studies performed on ultrasound guided injections were shown to be safe, but the interventions did not show significant improvements, even if studies are generally well-conducted, with a bias in the incomplete outcome data [51,53]. Ultrasound-guided tenotomy and dry needling needs to be studied in depth, through appropriate experimental study designs, due to the lower amount of evidence and the low quality of the studies produced.

This review has some limitations:—being a narrative review, it did not provide any statistical analysis of the included studies—but it presents the current situation on ultrasound use in the management of tendinopathies.

Future developments could be represented by 3D reconstruction of tendon movement during walking, as has been done for cerebral palsy children [60], and the identification of differences between healthy and pathological tendons.

### Limitations

Finally, it should be noted that there is an inherent bias in the methodological design of a literature review. Thus, the authors do not detail the score obtained in each of the articles in tables, which is characteristic of a systematic review

## 5. Conclusions

Ultrasound is a reliable, non-invasive and cost-effective imaging tool to assist the clinical diagnose of a tendinopathy. It is effective in the differentiation between a heathy tendon and an abnormal tendon; it causes more harm in the staging process of tendinopathy, but new techniques like UTC gave encouraging results for this process. Moreover, recently ultrasound has been combined with some treatments to guide the localization of the treatment only to the abnormal area of the tendon. Results are encouraging, but further research should investigate some of these techniques with high-quality studies.
